# Infodemic of covid-19 and repercussions on the mental health of the elderly from São Paulo

**DOI:** 10.1590/1980-220X-REEUSP-2021-0421en

**Published:** 2022-08-05

**Authors:** Jack Roberto Silva Fhon, Vilanice Alves de Araújo Püschel, Ricardo Bezerra Cavalcante, Fabiana Viana Cruz, Luan Nogueira Gonçalves, Wilson Li, Alice Regina Felipe Silva

**Affiliations:** 1 Universidade de São Paulo, Escola de Enfermagem, Departamento Médico-Cirúrgico, São Paulo, SP, Brazil.; 2 Universidade Federal Juiz de Fora, Faculdade de Enfermagem, Juiz de Fora, MG, Brazil.

**Keywords:** Aged, Coronavirus infections, Communication, Mental Health, Anciano, Infecciones por coronavirus, Comunicación, Salud mental, Idoso, Infecções por coronavírus, Comunicação, Saúde mental

## Abstract

**Objective:**

To characterize and identify depressive symptoms, anxiety, and stress associated with the COVID-19 Infodemic in the elderly from São Paulo.

**Method:**

Exploratory and cross-sectional study with the elderly in the capital of São Paulo who had internet access. The sociodemographic profile, the COVID-19 infodemic, depressive symptoms, stress, and anxiety were analyzed.

**Results:**

A total of 411 older people participated in the study. There was a predominance of women (76.4%), with higher education (57.9%), using private health services, and with little income variation. Older people were more exposed to news or information about COVID-19 on the internet (45.3%), followed by television (34.5%), and radio (11.4%). The average stress was 19.96 points; 33.1% had anxiety, and 39.7% had depressive symptoms. The greater the number of people living with the elderly, the greater the stress (p = 0.001) and anxiety (p = 0.02). The hours of exposure to information on the internet led to stress (p = 0.001), depressive symptoms (p = 0.02), and anxiety (p = 0.02) in the elderly.

**Conclusion:**

During the pandemic, exposure to information on the internet triggered anxiety, stress, and depressive symptoms in the elderly. The findings highlight the need for multi and interdisciplinary interventions to mitigate such repercussions on the elderly’s health.

## RESUMO

**Objetivo:**

Caracterizar e identificar sintomas depressivos, ansiedade e estresse associados à Infodemia de COVID-19 em idosos paulistanos.

**Método:**

Estudo exploratório e transversal com idosos da capital de São Paulo com acesso à internet. Foram analisados o perfil sociodemográfico, a infodemia de COVID-19, os sintomas depressivos, o estresse e a ansiedade.

**Resultados:**

Participaram 411 idosos. Predomínio do sexo feminino (76,4%), com ensino superior (57,9%), do uso de serviço de saúde privado e baixa variação na renda. Os idosos foram mais expostos a notícias ou informações sobre COVID-19 pela internet (45,3%), seguida pela televisão (34,5%) e pelo rádio (11,4%). A média de estresse foi 19,96 pontos; 33,1% apresentaram ansiedade e 39,7% sintomas depressivos. Quanto maior o número de pessoas que vivem com o idoso maior o estresse (p = 0,001) e a ansiedade (p = 0,02). As horas de exposição a informações pela internet levou a estresse (p = 0,001), sintomas depressivos (p = 0,02) e ansiedade (p = 0,02) do idoso.

**Conclusão:**

Durante a pandemia, a exposição às informações pela internet desencadeou ansiedade, estresse e sintomas depressivos nos idosos. Os achados evidenciam a necessidade de intervenções multi e interdisciplinares para mitigar tais repercussões na saúde dos idosos.

## RESUMEN

**Objetivo:**

Caracterizar e identificar síntomas depresivos, ansiedad y estrés asociados a la Infodemia COVID-19 en adultos mayores de São Paulo.

**Método:**

Estudio exploratorio y transversal con adultos mayores de la capital paulista con acceso a internet. Fueron analizados perfil sociodemográfico, infodemia de COVID-19, síntomas depresivos, estrés y ansiedad.

**Resultados:**

Participaron 411 adultos mayores. Predominio del sexo femenino (76,4%), con estudios superiores (57,9%), usuarios de servicios de salud privados y baja variación de ingresos. Los adultos mayores estuvieron más expuestos a noticias o información sobre el COVID-19 en internet (45,3 %), seguida de la televisión (34,5 %) y la radio (11,4 %). El estrés promedio fue de 19,96 puntos; el 33,1% tenían ansiedad y el 39,7% tenían síntomas depresivos. Cuanto mayor era el número de personas que vivían con los adultos mayores, mayor era el estrés (p = 0,001) y la ansiedad (p = 0,02). Las horas de exposición a la información en internet generaron estrés (p = 0,001), síntomas depresivos (p = 0,02) y ansiedad (p = 0,02) en los adultos mayores.

**Conclusión:**

Durante la pandemia, la exposición a información en internet desencadenó síntomas de ansiedad, estrés y depresión en los adultos mayores. Los hallazgos destacan la necesidad de intervenciones multi e interdisciplinarias para mitigar tales repercusiones en la salud de los adultos mayores.

## INTRODUCTION

Since the beginning of 2020, humanity has been affected by the pandemic of the new coronavirus, called, in March of that same year, by the World Health Organization (WHO), COVID-19 (Coronavirus Disease 2019), which is a respiratory infection caused by the Severe Acute Respiratory Syndrome Coronavirus 2 (SARS-Cov-2), which emerged in China in late 2019^(1,2)^.

In Brazil, the first case was reported in São Paulo, on February 25, 2020, and after three months it was considered the most affected state in Brazil, with almost 86,000 confirmed cases and 6,500 deaths from COVID-19^(3)^. With the exponential spread of the virus and the strategies to contain it, there was also the rampant dissemination of news, making it difficult for people to discern the veracity of the information disclosed. Thus, the world was not only facing a pandemic, but also an Infodemic^(4)^ that could lead to mental health changes in the elderly.

The term infodemic is defined as the rapid dissemination of a high flow of information about a given subject, which may or may not be true. In cases of disclosure of untrue information, they are popularly known by the term fake news. Thus, concomitant with the COVID-19 pandemic, the world is facing a global epidemic of misinformation that represents a real threat to the population’s health. Therefore, it is not enough to inform, it is necessary to analyze and fight sources of misinformation^(5)^.

From this perspective, with the objective of establishing evidence-based tools and interventions for dealing with the Infodemic, in July 2020 the WHO promoted the first scientific conference on the subject and released four pillars for the management of the Infodemic, which are: monitoring information; strengthening literacy capacity in digital health and science; improving the quality of information, conducting fact analysis and peer review; and translating knowledge, not to distort information, with political and/or commercial interest^(6)^.

In Brazil, as an initiative to fight fake news associated with the COVID-19 pandemic, a website was created (https://coronavirus.saude.gov.br/), where the user is directed to information about the actions carried out by the Ministry of Health, to consult the information disclosed. Moreover, a channel called “Health without fake news” (https://www.saude.df.gov.br/saude-sem-fake-news) was made available, which is a phone number linked to the *Whatsapp*, in which it is possible for the population to collaborate in the fight against false information. Conflicting information and news are sent and then checked. Later, they are officially answered regarding their veracity^(7)^.

However, even in the face of the establishment of strategies to face the Infodemic, the accelerated and exorbitant dissemination of information about COVID-19 can have negative impacts on the health of the population in general, especially the elderly’s mental health. It should be noted that, in the context of the current COVID-19 pandemic, the elderly have been considered a population group with greater physical and mental vulnerability. Physical, as the aging process is associated with a reduction in the immune response and with the onset of chronic diseases and comorbidities, favoring complications in the face of infection by the virus. With regard to mental impairment, it is observed that older people are more susceptible to emotional distress, caused by social distancing, and excessive exposure to conflicting information and recommendations, disseminated by the various official and unofficial sources of information. The sum of these two aspects (physical and mental) can trigger loneliness, sadness, depression, stress, and anxiety^(8)^.

An epidemiological study developed in China during the pandemic, with a sample of 1,556 elderly people, highlighted that 37.1% of the elderly had symptoms of anxiety and depression. The authors mentioned that limited access to mental health services and quarantine significantly increased social isolation, loneliness and, consequently, depression rates^(9)^.

A study developed by the Instituto de Psiquiatria do Hospital das Clínicas, Faculdade de Medicina da Universidade de São Paulo (HCFMUSP), with the objective of establishing the prevalence of behavioral and psychological symptoms in psychogeriatric patients in response to the COVID-19 pandemic, showed that 60% of the elderly evaluated reported psychiatric or psychological suffering in the face of the pandemic scenario. Among the most frequent symptoms, dysphoric/mixed symptoms (such as irritability and anger), anxiety and depression (lack of interest, low mood, and severe pessimism) stood out. When analyzing patients with a previous neurocognitive disorder, it was found that 64.3% of the participants reported depression and dysphoria^(3)^.

Given these findings, it is possible to consider that the outbreak of COVID-19 had a negative impact on the elderly’s mental health. Therefore, an in-depth investigation of the elderly’s mental health and the possible associated factors is required. Therefore, this study aimed to characterize and identify depressive symptoms, anxiety, and stress associated with the COVID-19 Infodemic in older people from São Paulo.

## METHODS

### Design of Study

This is a descriptive, exploratory, and cross-sectional study carried out in the city of São Paulo. This study is part of an International Multicenter Project entitled “COVID-19 Infodemic and its repercussions on the mental health of the elderly: a multicenter study in Brazil, Portugal, Chile, Peru, and Mexico”. The study was guided by the recommendations from Strengthening the Reporting of Observational Studies in Epidemiology (STROBE) and Checklist for Reporting Results of Internet E-Surveys (CHERRIES).

### Population

The study population consisted of elderly people. The sample definition was estimated by city, considering the elderly population, using the formula: n = N.Z^2^.p.(1–p)/Z^2^.p.(1–p). + e^2^. (N–1), where “n” is the calculated sample, “N” is the population, “Z” the standardized normal variable associated with the confidence level, “p” the true probability of the event [P = (1-P) = 0.5, maximum variation assumption], and “e” the sampling error, using a sampling error of 5% and a confidence level of 95%. Thus, in São Paulo (capital), the sample calculated was of 384 elderly people.

The inclusion criteria were: people aged 60 years or older, living in the city of São Paulo, with the ability to read and complete the instruments autonomously and online and who had access to e-mail, social networks and/or phone. The failure to fill in all the information was considered as an exclusion criterion.

Because of the social distancing due to the COVID-19 pandemic, the researchers could not have access to the elderly in person. Thus, as the contacts of elderly people were identified by the researchers, they were asked to indicate other elderly people or to forward the invitation to their respective contacts, using the virtual snowball technique to reach the sample number.

### Data Collection

Data collection was carried out from July 2020 to January 2021 through the web-based survey, with average filling time of 20 minutes. Before starting to collect the information, a pilot test was carried out to identify deficiencies and improve the instrument functionality. The link to access the survey was public, divided in two pages containing mandatory questions, and disseminated through social medias such as Facebook, Instagram, WhatsApp, among others. The Office of Vice-Dean for Culture and Extension Affairs at USP was also asked to disclose the research through the USP 60+ Program.

For data collection, the instruments described below were used.

Sociodemographic profile: obtaining data on sex (male and female), age (complete years), marital status (with and without a partner), education (years of study), people who live with the elderly person, people who depend on the elderly person’s income, use of health services, and income change during the pandemic.

Infodemic on COVID-19: identification of time data, knowing the number of hours that the elderly have browsed social media, how much time they have spent watching television and listening to the radio daily, and how they have obtained information, whether through social media (Facebook, WhatsApp, Instagram, among others) or television and radio.

Perceived Stress Scale: developed and validated for Brazilian Portuguese to measure the perceived stress of the elderly. The elderly were asked about their feelings and thoughts during the previous month, indicating how often they had felt in a certain way. The scale consists of 14 questions with five possible answers, with scores ranging from 0 to 56 and cut-off points are not suggested. The higher the score, the greater the person’s perception of stress^(10)^.

Geriatric Anxiety Inventory: validated for Brazilian Portuguese, assesses anxiety in the elderly. The scale consists of 20 questions with dichotomous answers (agree/disagree), according to the statements presented. The scale has a score of 8 as a cutoff point to indicate the presence of generalized anxiety, categorizing the elderly “with” and “without anxiety”^(11,12)^.

Geriatric Depression Scale: developed originally with 30 questions^(13)^ and later validated for Brazilian Portuguese in its 15-question version^(14)^. It is an instrument that aims to assess depression in the elderly population. The scale used presents dichotomous answers (yes and no), in which “yes” and “no” range from 0 to 1 point, depending on the question. The scale has a cut-off point of 5/6 points to categorize the elderly with and without the presence of depressive symptoms^(14)^.

### Data Analysis and Treatment

Data were stored in a spreadsheet of software Microsoft Excel^®^ and were analyzed using the software Statistical Package for the Social Sciences – SPSS v. 25.0. Descriptive statistics were used to identify measures of central tendency and dispersion for numerical variables and proportions for categorical variables.

In the analysis, multiple linear regression was used, having as an outcome the indication of stress, depressive symptoms, and anxiety, to identify the associations between demographic variables, such as sex, age, marital status, and number of people who lived with the older person. In the final model, data about the Infodemic were also included, such as: time of exposure to information on social networks, television and radio, and how the elderly obtained information about the pandemic. For the test, 5% significance level was considered.

### Ethical Aspects

The study was approved by the National Research Ethics Committee (*CONEP*), according to opinion No. 4.134.050, in 2020, and developed in accordance with Resolution 466/12.

The elderly participants, when accessing the link, were first directed to the digital Free and Informed Consent Form (FICF), where they could read the study objective, time to complete it, and acceptance or not to participate in the study. The acceptance or not to participate in the study was automatically registered in the database generated by the web-based survey. This bank will remain stored for a period of five years under the protection of the study coordinator.

## RESULTS

A total of 411 elderly people participated in the study, predominantly women and aged between 60 and 69 years. They had a partner and higher education. As for the number of people, 1.58 lived with the elderly and 1.65 depended on their income. Most of the elderly were retired, used a private health service and had no change in income during the pandemic (Table 1).

**Table 1.** Sociodemographic characterization of the elderly living in the city of São Paulo. Sao Paulo, 2021.

Variable	Category	n (%)	%	Mean (=SD)
Sex	Female	314	76.4	
	Male	97	23.6	
Age	60 – 69	287	69.8	67.41 (6.82)
	70 – 79	97	23.6	
	80 or more	27	6.6	
Marital status	With a partner	232	56.4	
	Without a partner	179	43.6	
Education	No studies	12	2.9	
	Elementary School	91	22.1	
	High school	70	17.1	
	University education	238	57.9	
People who live with the elderly				1.58 (1.37)
People dependent on the elderly’s income				1.65 (3.79)
Origin of the elderly’s income	Retirement	319	77.6	
	Job	114	27.7	
	Government benefits	12	2.9	
	Dependent on others	17	4.1	
Use of health services	Private	191	46.5	
	Private/Brazilian Public Health Service (SUS)	123	29.9	
	SUS	96	23.4	
	None	1	0.2	
Change in income during the pandemic	No	292	71.1	
	Decreased	109	26.5	
	Increased	10	2.4	

Regarding exposure to the media, it was found that the elderly were more exposed to news or information about COVID-19 on the internet (45.3%) and then on television (34.5%) and radio (11.4%) (Table 2).

**Table 2.** Exposure to information about the COVID-19 pandemic by the elderly living in the city of São Paulo. Sao Paulo, 2021.

Variable	Category	n (%)	Mean (=SD) – Min – Max
Exposure to television news	None	85 (20.7)	2.86 (3.82) 0 – 24
	Few times	99 (24.1)	
	Sometimes	85 (20.7)	
	Frequently	142 (34.5)	
Exposure to Internet news	None	44 (10.7)	2.93 (4.13) 0 – 24
	Few times	97 (23.6)	
	Sometimes	84 (20.4)	
	Frequently	186 (45.3)	
Exposure to radio news	None	257 (62.5)	0.76 (2.19) 0 – 24
	Few times	67 (16.3)	
	Sometimes	40 (9.7)	
	Frequently	47 (11.5)	

As for the evaluation of the elderly, the average perceived stress was 19.96 points; 33.1% had anxiety and 39.7% had depressive symptoms (Table 3).

**Table 3.** Stress, depressive symptoms and anxiety, during the pandemic, of the elderly living in the city of São Paulo. Sao Paulo, 2021.

Variables	n	%	Mean (=SD)
Stress			19.96 (10.03)
Anxiety			5.76 (5.63)
No anxiety	275	66.9	
With anxiety	136	33.1	
Depressive symptoms			5.41 (2.61)
No symptoms	248	60.3	
With symptoms	163	39.7	

The means most used by the elderly participating in the research to obtain information were television (80.3%), social media such as *Whatsapp* (49.1%), internet pages (44.3%) and Facebook (33.3%).

**Table 4.** Means of obtaining information about the pandemic by the elderly living in the city of São Paulo. Sao Paulo, 2021.

Obtaining the information	n	%
Television	330	80.3
Whatsapp	202	49.1
Websites	182	44.3
Facebook	137	33.3
Newspapers or magazines	98	23.8
Radio	89	21.7
Youtube	84	20.4
Instagram	37	9.0
Twitter	23	5.6
Telegram	7	1.7
Others	14	3.4

In the regression model, it was identified that the greater the number of people living with the elderly, the greater the stress (p = 0.001) and anxiety (p = 0.02). When comparing the hours of exposure to information on the internet, it was found that there is an increase in stress (p = 0.001), depressive symptoms, (p = 0.02) and anxiety (p = 0.02) of the elderly (Table 5).

**Table 5.** Association between stress, depressive symptoms, and anxiety with the study variables in the elderly living in the city of São Paulo during the pandemic. Sao Paulo, 2021.

Variables	Stress		Depressive symptoms		Anxiety	
	Beta	P	Beta	P	Beta	P
Sex (Female)	1.24	0.29	0.52	0.08	0.50	0.46
Age	–0.11	0.13	0.01	0.36	–0.02	0.51
Marital status (with partner)	–1.38	0.19	–0.08	–0.54	–0.35	0.55
Number of people living with the elderly	1.20	0.001	0.15	0.11	0.47	0.02
Hours of exposure to internet news	0.47	0.001	0.08	0.02	0.18	0.02
Hours of exposure to television news	–0.01	0.92	0.02	0.54	0.07	0.36
Hours of exposure to radio news	0.003	0.98	–0.02	0.66	0.07	0.56

## DISCUSSION

In this study, it was identified that television was the means most used by the elderly to obtain information about the pandemic, followed by the use of the application of Whatsapp and websites. However, there was a statistically significant association between hours of internet exposure and self-reported anxiety, stress and depressive symptoms in the elderly. It was also found that the greater the number of people who live with the elderly, the greater the stress and anxiety.

Of the 411 elderly people from São Paulo who participated in the study, there was a predominance of woman, corroborating data disclosed by the city of São Paulo in 2020^(15)^, that about 60% of the elderly in São Paulo are women, and this finding is common in other studies^(4,9,16)^. This occurrence may be associated with the greater acceptability of women to participate in studies and may also be associated with greater female longevity, greater frequency of self-care and the use of health services^(16)^. Compared to a study carried out in the city of Recife^(16)^, there was divergence regarding the predominance of marital status, which showed that the majority consisted of elderly people living without partners, while the sample of the present study consists mostly of elderly people living with partners, in accordance with the 2010 census^(15)^, in which only 14.9% of the elderly lived alone.

The research shows that just over half of the sample of elderly people in São Paulo have higher education and almost a third remain employed, an aspect that differs from the report disclosed by the city hall of São Paulo in 2019^(17)^, showing that about 44% of the employed elderly had no schooling or had not finished elementary education, and that less than 1/4 of the employed elderly had higher education. Research data lead to the assumption that the snowball effect has contributed to reaching elderly people with similar sociodemographic characteristics, although it also highlights the effects of the COVID-19 infodemic in elderly people with higher education. Furthermore, the city of São Paulo has public and private universities that have different accessible undergraduate programs, which increases a person’s educational level.

There was a predominance of the use of private health services and the main source of income is retirement, showing that those who participated in the study belong to a more educated group, thus allowing inferring that its members have a higher and more stable income (retirement), which allows them to pay for private health care, a fact that was already observed in a study carried out in 2006^(18)^, in which the variables of education, income, and private health are directly linked.

It was identified that 1/3 of the elderly have anxiety, 2/5 have depressive symptoms and the average stress was 19.96 points. In this case, there are similarities between the data obtained in this study and a study carried out in China^(9)^. However, when compared to the one developed by the HCFMUSP Institute of Psychiatry^(3)^ with elderly people who had pre-existing neurocognitive and psychiatric disorders, it was found that about 60% of the sample had psychiatric or psychological distress related to COVID-19. It can be inferred that the pre- existence of psychiatric disorders is a possible risk factor for the development of anxiety, stress, and depressive symptoms.

In our study, the elderly responded that living and coexisting with family members causes them to suffer stress and anxiety. This can be triggered by the fear of being infected or infecting family members, which could increase the chain of transmission and illness, with the presentation of mild and even severe symptoms that require hospitalization^(19)^. Research on infectious outbreaks revealed that populations suffered negative impacts on the psychological aspect in the short, medium and long term^(20)^.

It is understood that, with social distancing, the contact that the elderly had with their family and friends was even lower, causing feelings of loneliness and aggravation of anxiety and stress, generated by the uncertainty of what was to come, in addition to fear and concern for their family and friends, which can generate a feeling of incapacity and uselessness that contributes to the emergence of psychological changes^(21)^.

To avoid anxiety and stress, the WHO recommended keeping contact with family and friends through digital means, having a good diet, regulating sleep, maintaining an exercise routine, as well as being aware of their own demands and seeking information about COVID-19 from reliable sources^(22)^.

Regarding the exposure to means of communictaion, the older people were more exposed to news or information about COVID-19 on the internet (45.3%), followed by television (34.5%), and radio (11.4%). This finding can be associated with the dissemination of fake news on social media, as it is the platform where there is greater dissemination and exposure to information about the pandemic, with an increase in false or misleading news^(23)^.

As the COVID-19 pandemic is a situation never experienced by the Brazilian and world population on such a scale, and due to the greater ease of access to information vehicles, there was an increase of information on the internet, especially through social media, making the control of the amount and authenticity of the news broadcast more difficult, favoring the phenomenon of the COVID-19 Infodemic^(24)^, causing fear, anxiety, and depressive symptoms identified in this study.

As for obtaining information related to the pandemic, it was found that television is the main medium used by the population studied, followed by Whatsapp. The other sources of information also used are the Facebook, by a third of the participants, in addition to newspapers and magazines, radio and YouTube, by about a quarter of the participants. A study carried out in Canada with the elderly showed that the beginning of the COVID-19 pandemic led to an increase in the use of Facebook (p = 0.008), television (p = 0.006) and YouTube (p = 0.04) for information; and, for entertainment or leisure, streaming services such as Netflix (p = 0.01)^(25)^.

The Infodemic, aggravated in the context of the COVID-19 pandemic, presents itself as a dangerous cycle, for accelerating and disseminating disinformation, as well as negatively affecting the population’s mental health, especially the elderly’s^(23,24)^. There are different explanations for the numerous sharing of fake news, the main ones being the absence of technical-scientific knowledge and insufficient critical analysis regarding the veracity of the content transmitted^(26)^.

Studies highlight the negative effects of the pandemic on general population feelings. The increased use of the internet and social media suggests that, for some, such use can be a coping mechanism to fight feelings of isolation related to long-term social distancing. However, in light of the negative effect, constant use can affect mental health, further exacerbating negative feelings in the long run^(27)^.

Mental health in the population has been affected during the COVID-19 pandemic, especially that of the elderly, who will need care. In Brazil, in recent decades, the network of specialized services, such as the Psychosocial Care Centers (*CAPS*), has been enhanced, but training of health professionals and those working in this area to provide care is still scarce^(28)^, even in São Paulo, which is the richest state in Brazil.

The health and nursing team is present at all stages of a person’s life, having as a fundamental role, in addition to the provision of care and well-being, the dissemination of information and knowledge. In this regard, it plays an important role in health education and should include in its actions care for the information that can be disseminated, to mitigate the Infodemic, especially in times of a pandemic. Misinformation and the fake news, so widely disseminated today, are issues that also need to be considered as a central point in health care.

Governments, on their turn, shall invest heavily in health campaigns to fight fake news, adopting strategies for disseminating health information adapted to the context, groups and levels of education^(29)^, especially in the communication vehicles identified in the study, as they will be accessed by the elderly. Furthermore, institutions such as the WHO have disseminated strategies to mitigate the infodemic in the population.

It is important to engage managers of social media platforms to control messages delivered^(30)^, using artificial intelligence, to fight fake news, directing user search algorithms to reliable sources based on scientific evidence.

Health professionals, on their turn, need to seek continuous updating, promote dialogue to understand people’s perceptions and the reasons behind their practices, develop and share educational material based on scientific evidence^(30)^.

There is also a need for studies that seek to understand the impacts that the dissemination of false information or even excess information can generate on health, especially in the elderly.

The main limitation of this study is associated with data collection, since due to the need for social isolation imposed by the COVID-19 pandemic, data were collected through the dissemination of the invitation to respond to the web-based survey through social media. This possibly favored showing results from more educated and better-resourced elderly people; in addition, the snowball technique was used, which tends to make the sample homogeneous. For future studies, with the advancement of vaccination in the country, longitudinal multicentric research is suggested to evaluate the impact of the dissemination of information related to the number of infected people and deaths and its impact on the mental health of these elderly people, as well as to expand the collection to reach the elderly from different social strata, from different geographic regions, aiming to portray the Brazilian reality.

## CONCLUSION

In the study, there was a predominance of women; aged between 60 and 69 years, with a mean of 67.41 years; with a partner; with higher education; and retired people. The average number of people who live with the elderly person was 1.58 and that of people who depend on this income was 1.65 people. As for the use of health services, private service predominated and there was no change in income during the pandemic.

During the pandemic, the elderly showed anxiety, stress, and depressive symptoms. There was an association between the number of people who lived with the elderly and stress and depressive symptoms. Moreover, exposure to information on the internet triggered anxiety, stress, and depressive symptoms in the elderly.

The study brings contributions to practice, teaching, and research. For practice, the findings of the study highlight the need for multi and interdisciplinary interventions to mitigate such repercussions on the elderly’s health. For teaching, it is important that in the training of health professionals, in undergraduation and permanent education, the repercussions caused by the pandemic on the elderly’s mental health are the focus of an interdisciplinary approach. For the research, further studies are required to understand the impacts generated by the excess of information to the elderly and how this could be mitigated by health professionals, especially nurses, considering that their health education actions shall include care to the elderly as a person who experiences a new phenomenon that is present in everyday life, the Infodemic.
